# Effect of Lockdown on Diabetes Care During the COVID-19 Pandemic: Result of a Telephone-Based Survey Among Patients Attending a Diabetic Clinic in Northern India

**DOI:** 10.7759/cureus.18489

**Published:** 2021-10-05

**Authors:** Madhur Verma, Priyanka Sharma, Atul Chaudhari, Meenakshi Sharma, Sanjay Kalra

**Affiliations:** 1 Department of Community and Family Medicine, All India Institute of Medical Sciences, Bathinda, IND; 2 Department of Community Medicine, Vardhaman Mahavir Medical College (VMMC) & Safdarjung Hospital, New Delhi, IND; 3 School of Public Health, Post Graduate Institute of Medical Education and Research, Chandigarh, IND; 4 Endocrinology, Bharti Hospital, Karnal, IND

**Keywords:** lockdown, diabetes care, pandemic, medication adherence, dietary pattern

## Abstract

Background

The coronavirus disease 2019 (COVID-19) pandemic has aggravated the demand for diabetes care due to restrictive measures like the lockdown affecting access to healthcare services. The current study was conducted to assess the changes in medication compliance, dietary pattern, and glucose monitoring during the lockdown period as compared to the pre-lockdown period among patients living with type 2 diabetes mellitus* *(T2DM) attending a diabetes clinic in northern India.

Methods

This cross-sectional study was conducted between May and July 2020. Information regarding the sociodemographic and clinical profiles of the patients like age, sex, income, qualification, family history of diabetes, history of smoking and alcohol, type of treatment, co-morbidities, drug adherence for T2DM, changes in the pattern of diet, physical activity, blood glucose monitoring, and drug usage during and before the lockdown was collected through telephonic interviews using a structured tool. Descriptive analysis was performed, and the chi-square and Wilcoxon sign ranks tests were used to see the association between variables.

Results

A total of 260 patients were enrolled in the study. A higher proportion of males reported a decrease in the consumption of cereals (13.9%), eggs (56.5%), and meat and fish (92.7%) and an increase in water intake (25.8%) while a higher proportion of females reported no change in physical activity levels (77.2%) during the lockdown against pre-COVID times. There was a significant improvement in medication adherence and glycemic control during the lockdown period as compared to the pre-lockdown times.

Conclusion

More time for self-care, adequate counseling about glycemic goals, and knowledge of self-monitoring of blood glucose levels helped the majority of patients in adopting a healthy lifestyle and achieve better glycemic control during the COVID-19 lockdown.

## Introduction

Approximately 422 million people are living with diabetes around the world, with a majority of them belonging to low and middle-income countries [1]. The prevalence of diabetes among Indian adults is about 8.9% [2], and India is considered to be the diabetes capital of the world [3]. Diabetes is considered to be one of the most psychologically and behaviorally demanding chronic diseases. It requires strict lifestyle and dietary modifications, with frequent self-monitoring of blood glucose and adherence to the prescribed medications [4].

This demand was further aggravated during the coronavirus disease 2019 (COVID-19) pandemic while it has caused significant morbidity and mortality around the world. India is amongst the worst-hit countries in terms of the number of COVID-19 cases [5]. It has overwhelmed the existing health system, and most of the resources have been diverted towards pandemic mitigation. Due to the lack of any specific treatment during the initial period, only non-pharmacological interventions like physical distancing were seen as effective interventions to flatten the curve of the pandemic. Physical distancing in populous countries like India was enforced using a lockdown, where the government used administrative powers to implement physical distancing, and people were obligated to stay in their houses [6]. Lockdowns restricted the movements on a large scale, as they could slow the disease transmission by checking contact between people [7-8].

But such strict measures also negatively affected social and economic life. Lockdown severely restricted access to health care facilities for the majority of people in need. As a result, patients living with health conditions other than COVID-19 (both acute and chronic) were devoid of requisite health care services. In fact, comorbidities (diabetes, high blood pressure, heart disease, obesity, lung problems, cancer, and age more than 60 years) have been associated with a higher risk of morbidity and mortality due to COVID-19 [9]. Lockdown restrictions have affected the pharmacological and non-pharmacological management of non-communicable diseases like diabetes mellitus. This can be attributed to decreased accessibility to doctors consultation, including follow-up visits, and inappropriate drugs therapy, apart from lack of adequate counseling services, decreased medication adherence, changes in dietary patterns, reduction in physical activity levels, and decreased social interaction leading to mental health problems [10].

Medication non-adherence is common among diabetics, and it is one of the leading public health challenges [11]. In a resource-poor country like India with low literacy levels and restricted access to healthcare facilities, the prevalence of medication non-adherence is much more common [12]. Poor adherence leads to poor glycemic control, increased rates of complications leading to higher rates of morbidity and mortality, and higher out-of-pocket expenditure [13]. The same has been expected during the lockdown period, when sedentary lifestyles, poor dietary habits, and sleep deprivation prevailed, which are potentially modifiable risk factors for poor glycemic control in people with diabetes. Hence, it has been hypothesized that lockdown during the COVID-19 pandemic may be associated with poor glycemic control in people with diabetes. Isolated studies in type 1 diabetes individuals have conflicting reports of worsening or no impact of lockdown period on glycemic control [14-15]. Previous studies from India and abroad have depicted mixed results in response to this research question for type 2 diabetes mellitus (T2DM). Rastogi A et al. reported an overall improvement of glycemic control during COVID-19 lockdown [16], but a majority of studies have reported poor glycemic control post-lockdown [17]. Alshareef R et al. reported reduced compliance to medications and healthy lifestyle behaviors among T2DM patients post lockdown in Saudi Arabia [18]. Hence, the current study was conducted to assess the changes in diabetes management during the lockdown period as compared to pre-lockdown among patients living with T2DM and attending a diabetes clinic in northern India.

## Materials and methods

Study design and duration

A cross-sectional study design was used to conduct this study and data collection was done between May and July 2020.

Study setting

The study was conducted among patients attending a private endocrine clinic in district Karnal in the state of Haryana, from the northern part of India. It is operated by super-specialist clinicians. Apart from therapeutic care for various endocrinological disorders, including diabetes, the clinic also offers pre and post-consultation counseling sessions, monitoring of blood sugar levels, and screening for micro and macro-vascular complications related to T2DM. The clinic also maintains complete demographic and disease-related details in an electronic case record system. Similar to the whole country, the health system in Karnal was also affected due to the lockdown that was enforced in March 2020 and was further released in a phasic manner [6].

Study population

For the purpose of this study, patients (> 18 years) living with T2DM for more than one year with updated contact details were approached while we excluded patients living with type 1 diabetes, the absence of a facility for self-monitoring of blood glucose during the lockdown period, incomplete contact details, or a confirmed case of COVID-19 infection.

Sample size and sampling method

The sample size was calculated using Epi Info (version 6.04d), developed by the Center for Disease Control, Atlanta, USA, and the WHO. We considered the prevalence of low treatment adherence to diabetes treatment at 25% [19], with 95% confidence limits, 7.5% precision, and a design effect of 2. The calculated sample came to be 257, and hence a representative sample of 260 people living with T2DM was selected.

We adopted a systematic random sampling technique to recruit study participants. Using the hospital records, every fifth patient as per inclusion criteria was approached telephonically. We called a total of 300 patients and out of them, 275 responded and agreed to the interview. Verbal informed consent was obtained from all study participants before the interview. The consent form had two parts: information for the participant and the actual consent form. Confidentiality of the data was ensured to the participants. After data cleaning, we ultimately included 260 participants who completed the interview and their responses were included in the final analysis.

Data variables

Data were collected with the help of a structured tool by the authors of the study through telephonic interviews with the study participants. The calls were made by the same study investigator who was trained before the initiation of the study. The tool consisted of three parts. Part A included information pertaining to the sociodemographic and clinical profiles of the patients like age, sex, income, qualification, marital status, residence, family history of diabetes, history of using tobacco products and alcohol, type of treatment, and co-morbidities. We asked for the last values of the blood glucose levels as per their records, within the last three months just before the lockdown and during the lockdown. Part B included a survey instrument to assess drug adherence for T2DM. For this, we used a pre-tested, validated, structured five-item MARS (Medication Adherence Report Scale, ©Professor Rob Horne) scale [20]. It is a short version of the original 10-item scale and has depicted reliable psychometric properties comparable to the original version of MARS. MARS‐5 shows promise as an effective tool for assessing adherence, identifying patients with low adherence and the specific types of nonadherence behaviors (eg, forgetting, deliberately missing doses), and is available freely in the public domain. Part C collected data regarding changes in the pattern of participant’s diet, physical activity, blood glucose monitoring, and drug usage during and before the lockdown.

Statistical analysis

The data were recorded in Microsoft Excel and analyzed using SPSS version 21 (IBM Corp, Armonk, NY). Post-data cleaning and coding, descriptive analysis was performed in the form of frequency and proportions for categorical variables. The chi-square test was used to test the association between the sociodemographic variables to highlight any gender-based differences. Drug adherence using MARS-5 was recorded on a five-item Likert scale. The responses during the lockdown and pre-lockdown period were compared using the Wilcoxon sign ranks test. Changes in diet, physical activity, and stress patterns during and before lockdown were coded as paired ordinal/ranked data as ‘increased,’ ‘decreased,’ or ‘no change.’ All p-values <0.05 were considered statistically significant.

Ethics

The study was conducted within the boundaries of the Helsinki Declaration. Informed consent was obtained from all the study participants. The privacy and confidentiality of the data were ensured.

## Results

The telephonic interviews were completed for 260 patients having T2DM. The mean age of the study participants was 51.5 + 11.30 years. The differences in the mean fasting blood glucose levels in the pre-lockdown period (131.96 + 97.52 mg/dl), and during the lockdown period (119.2+74.1 mg/dl) were statistically significant (p-value 0.015). However, the mean post-prandial blood sugar levels did not vary significantly (p-value 0.195) before (184.95 + 122.95 mg/dl) and during the lockdown (174.5+92.1 mg/dl) period.

More than two-thirds of the study participants (71.1%, 185) were above the age of 45 years. The majority (61.2%) of the participants were males. The mean monthly income was around 45,000 INR. A significantly higher percentage of males were graduates or postgraduates and were employed in professional or semi-professional jobs as compared to females (p-value <0.01). More males were current alcohol and tobacco users compared to females (p-value <0.01). The most common co-morbidities were retinopathy and hypertension followed by neuropathy (Table 1). The prevalence of neuropathy was found to be significantly higher among women as compared to men (p-value 0.04). There were no significant differences in age, residence, family history of diabetes mellitus, type of treatment, and co-morbidities except neuropathy amongst males and females (Table 1).

**Table 1:** Demographic profile of the study population (N=260)

	Female	Male	Total	Chi-square (p-value)
Total	101 (100)	159 (100)	260 (100)	
Age in years				0.3 (0.58)
<45 years	31(30.7)	54(34.0)	85(32.7)	
>45 years	70(69.3)	105(66.0)	175(67.3)	
Education				31.8 (0.00)
Less than high school	41(40.6)	17(10.7)	58(22.3)	
High school & above	60(59.4)	142(89.3)	202(77.7)	
Earning money				169.1 (0.00)
Yes	6(5.9)	140(88.1)	146(56.2)	
No	95(94.1)	19(11.9)	114(43.8)	
Residence locality				0.82 (0.36)
Rural	49(48.5)	68(42.8)	117(45)	
Urban	52(51.5)	91(57.2)	143(55)	
Socio-economic status				3.87 (0.27)
1 (Highest class)	37(36.6)	72(45.3)	109(41.9)	
2	43(42.6)	66(41.5)	109(41.9)	
3	17(16.8)	15(9.4)	32(12.3)	
4	4(4.0)	6(3.8)	10(3.8)	
5 (lowest class)	0	0	0	
Marital status				0.06 (0.80)
Married	96(95.0)	150(94.3)	246(94.6)	
Single/widowed/divorced	5(5.0)	9(5.7)	14(5.4)	
Substance abuse				
Smoking	0	26(16.4)	26(10)	18.5 (0.00)
Alcohol	0	44(27.7)	44(16.9)	34.8 (0.00)
Family history of diabetes				1.95 (0.16)
Yes	45(44.6)	85(53.5)	130(50)	
No	56(55.4)	74(46.5)	130(50)	
Duration of diabetes				0.105 (0.746)
<5 years	23(22.8)	39(24.5)	62(23.8)	
>5 years	78(77.2)	120(75.5)	198(76.2)	
Type of treatment				8.23 (0.016)
Oral hypoglycemic agents	64(63.2)	131 (82.4)	185 (71.2)	
Insulin	11(10.9)	17 (10.7)	28 (10.8)	
Both oral drugs and insulin	26 (25.7)	21 (13.2)	47 (18.1)	
Comorbidities				
Hypertension	25(24.8)	25(15.7)	50(19.2)	3.24 (0.07)
Stroke	3(3)	2(1.3)	5(1.9)	0.96 (0.32)
Myocardial infarction	10(9.9)	7(4.4)	17(6.5)	3.05 (0.80)
Neuropathy	11(10.9)	7(4.4)	18(6.9)	4.03 (0.04)
Retinopathy	19(18.8)	20(12.6)	39(15)	1.88 (0.17)
Kidney disease	4(4)	4(2.5)	8(3.1)	4.32 (0.51)
Other comorbidities	2(2)	1(0.6)	3(1.2)	0.98 (0.32)

Table 2 showed that a significantly higher percentage of males reported a decrease in the consumption of cereals, eggs, meat, and fish and an increase in water intake as compared to females during the lockdown against the pre-lockdown times (p-value <0.05). A higher proportion of females as compared to males reported no change in physical activity levels during the lockdown (p-value 0.002). No significant gender difference was observed in changes in other dietary products intake, stress, and glucose monitoring.

**Table 2:** Diet and physical activity (self-care) of the diabetic patients during the lockdown

	Female	Male	Total	P Value
Total	n=101	n=159	n=260	
Dietary parameters				
Cereals				
Increase	2 (2)	5 (3.2)	7(2.7)	0.047
Decrease	4 (4)	22(13.9)	26(10)	
No change	95(94)	131(82.9)	226 (86.9)	
Fruits				
Increase	32(31.7)	59(37.3)	91 (35.0)	0.628
Decrease	10(9.9)	13(8.2)	23 (8.8)	
No change	59(58.4)	86(54.4)	145 (55.8)	
Vegetables				
Increase	24(23.8)	41(25.8)	65 (25.0)	0.8
Decrease	5(5.0)	6(3.8)	11(4.2)	
No change	72(71.3)	112(70.4)	184 (70.8)	
Roots & tubers				
Increase	4(4.0)	14(8.8)	18(6.9)	0.26
Decrease	6(5.9)	12(7.5)	18(6.9)	
No change	91(90.1)	133(83.6)	224 (86.2)	
Dairy products (n=252)				
Increase	11(11.1)	12(7.8)	23(8.8)	0.07
Decrease	3(3)	16(10.5)	19(7.3)	
No change	85(85.9)	125(81.7)	210 (80.8)	
Pulses				
Increase	5(4.9)	9(5.7)	14(5.4)	0.4
Decrease	2(2)	8(5)	10(3.8)	
No change	94(93.1)	142(89.3)	236 (90.8)	
Eggs (n=55)				
Increase	0	2(4.3)	2(0.8)	0.001
Decrease	2(22.2)	26(56.5)	28 (50.9)	
No change	7(77.8)	18(39.2)	25 (45.5)	
Meat & fish (n=50)				
Increase	0	0	0	0.002
Decrease	7(77.8)	38(92.7)	45 (90.0)	
No change	2(22.2)	3(7.3)	5 (10.0)	
Water				
Increase	13(12.9)	41(25.8)	54(20.8)	0.04
Decrease	2(2)	2(1.2)	4(1.5)	
No change	86(85.1)	116(73)	202 (77.7)	
Other parameters				
Physical activity				
Increase	0	9(5.7)	9 (3.5)	0.002
Decrease	23(22.8)	58(36.5)	81 (31.2)	
No change	78(77.2)	92(57.9)	170 (65.4)	
Stress				
Increase	8(7.9)	25(15.7)	33 (12.7)	0.17
Decrease	1(1.0)	1(0.6)	2(0.8)	
No change	92(91.1)	133(83.6)	225 (86.5)	
Glucose monitoring				
Increase	19(18.8)	37(23.3)	56 (21.5)	0.647
Decrease	20(19.8)	27(17.0)	47 (18.1)	
No change	62(61.2)	95(59.7)	157 (60.4)	

Medication adherence was compared with the help of MARS-5, which recorded the response on a five-point Likert scale. For the purpose of this study, we recorded responses categorized as ‘never’ and ‘rarely,’ and ‘often’ and ‘always' as single variables due to the smaller number of responses. A higher proportion of respondents said that they never missed their medications during the lockdown while the proportion of people who stopped them for a while on their own did not change significantly during the lockdown as compared to the pre-lockdown normal days. There was also a significant improvement (p-value <0.05) in the medication adherence, as a smaller proportion of respondents missed out, or altered their doses, and forgot to take their medicines during the lockdown period and compared to the pre-lockdown times (Table 3).

**Table 3:** Compliance to medicine adherence related behavior during the lockdown among diabetic patients enrolled in the study using the MARS-5 tool MARS-5: Medication Adherence Report Scale *Wilcoxon sign ranks test

Parameters	Normal days			During Lockdown			p-value
	Never/rarely	Sometimes	Often/always	Never/rarely	Sometimes	Often/always	
I take less than instructed	183(70.4)	73(28.1)	4(1.5)	234(90.0)	24(9.2)	2(0.8)	<0.01
I stop taking it for a while	242(93.1)	15(5.8)	3(1.2)	244(93.8)	14(5.4)	2(0.8)	0.564
I miss out a dose	237(91.2)	18(6.9)	4(1.5)	244(93.8)	12(4.6)	4(1.5)	0.014
I alter the dose	243(93.5)	17(6.5)	0	259(99.6)	1(0.4)	0	<0.01
I forget to take it	232(89.2)	25(9.6)	3(1.2)	256(98.5)	4(1.5)	0	<0.01

Table 4 depicted that out of 75 study participants who were taking insulin, all of them were taking it regularly and the majority didn’t alter the insulin dose on their own during the lockdown. A higher proportion of respondents reused their insulin syringes more than five times. No significant gender differences were found in insulin usage during the lockdown period.

**Table 4:** Insulin usage of the diabetic patients during lockdown (n=75) Chi-square test

Parameters	Male (n=38)	Female (n=37)	Total	p-value
Taking insulin	38(100)	37(100)	75(100)	0.027
Self-alteration of dose				0.096
Always	1(2.6)	0	1(1.3)	
Sometimes	7(18.4)	5(13.5)	12(16.0)	
Never	30(78.9)	32(86.5)	62(82.7)	
Reuse insulin syringe				
<5	16(42.1)	16(43.2)	32(42.6)	0.92
>5	22(57.9)	21(56.8)	43(57.3)	

## Discussion

The COVID-19 lockdown has adversely affected the health of people around the world, but low- and middle-income countries (LMICs) are predicted to be the worst-hit [21]. Emergency services have been prioritized over routine care services in most countries. This scenario has jeopardized the management of chronic diseases including non-communicable diseases. The lockdown’s effect on the management of diseases like diabetes should be explored, as COVID-19 is not the last pandemic and the world has to be prepared for similar such unprecedented events with an equal or higher magnitude. With this rationale, the present study was conducted to provide insight into diabetes management in adverse conditions.

During the inception of the research question, we hypothesized that lockdowns will adversely affect the management of diabetes. However, we observed that a majority of the participants did not report any major change in their disease management. Indeed, the compliance with medicines and glycemic control was observed to be better during the lockdown though we did not explore the reasons for the same. Most of our respondents denied any changes in their dietary patterns. Consumption of cereals, dairy products, pulses, and water was not much affected during the lockdown as compared to their normal (pre-lockdown) days. One-fourth of our participants reported an increase in consumption of vegetables, and one-third reported an increase in their fruit consumption during the lockdown, similar to other studies conducted in different parts of the country [22]. Fruits and vegetables are important for boosting the immune system, which is helpful against COVID-19 and was promoted strongly on social media. The increased consumption can also be attributed to the decreased availability of packaged junk foods, closure of eating places during the lockdown, and fear of contracting the disease, which forced the people to a behavior change. However, there was a decrease in the consumption of eggs, meat, and fish by 50-90%. A plausible explanation for the same relates to the decreased availability of these items. Also, these food products are relatively costlier than the vegan items and the lockdown led to an economic crunch for many people, which may have forced them to spend frugally and limit the expenditure on eggs, meat, fish, and similar items, as we found wide variations in the income levels of the study participants. Further, various myths that associated the spread of COVID-19 with meat products were rampantly circulated on social media, further discouraging their consumption [23]. Significant gender differences were observed regarding the consumption of food items, with a larger proportion of males reporting a decrease in the intake of certain food products like cereals, eggs, meat, and fish during the lockdown.

One-third of our respondents reported a decrease in physical activity during the lockdown, similar to another study conducted in Rajasthan and Delhi [24]. However, this decrease was higher than reported in some other studies [18,25]. Khader MA et al. reported a higher decrease in daily physical activity levels, as the majority of their study respondents had one or the other co-morbidity and belonged to urban areas [17]. Fear of mortality in patients with multiple co-morbidities and residence in urban areas might have played a role in restricting the movement during lockdown amongst their study participants. These variations in physical activity levels, when compared to previous studies, can be attributed to male preponderance in our study, as this decrease in physical activity was significantly higher among the male respondents. A higher proportion of the males were gainfully employed and the majority of the females were housewives in the present study. Lockdown restrictions led to the closure of offices and other workspaces, leading to a decrease in physical activity among these working men. But housewives continued working at homes with little disruption of their daily schedule, thereby explaining the reasons for the lower proportion of females reporting a decrease in their daily physical activity. Another concept that can explain the decrease in physical activities at home is related to the walkability index. Many residential complexes don’t have any provision for physical activities in close proximity to the houses, and hence physical activity was observed to decrease, increase, or not change in different studies.

About 13% of the diabetic patients in the present study reported an increase in self-perceived stress during a lockdown. Though stress was not assessed quantitatively using a validated tool, as it may have increased the time duration of our interviews, we tried to assess the levels of stress among the respondents. Our findings were in coherence with the findings of another study from Kerala [22]. But this proportion was much lower than another study from Delhi that reported the psychological effect of lockdown in about 87% of patients. Ghosh et al [26], however, reported that only 10% of the participants reported feeling low. The variations in the proportion of psychologically affected individuals in this study could be due to the timing of the interviews and the pre-existing social, medical, behavioral, and environmental conditions that may affect baseline stress levels.

We observed that around one-fifth of our study participants reported a decrease while the majority of them depicted no change in the frequency of blood glucose monitoring during the lockdown period. This was similar to the finding of Sankar P et al. where the majority reported no change [22]. However, AL Agha EA et al. [27] and Ghosh A et al. [26], reported a major decrease in the frequency of blood glucose monitoring. In rural areas and small cities, most people rely on laboratories, which were closed during the lockdown, for their blood glucose estimation. So, if people living with diabetes are taught to self-monitor their blood glucose levels and are motivated to do it on a regular basis and maintain a glycemic chart, this activity can lead to a significant improvement in glycemic control. The majority of our participants never ran out of medicine, similar to the findings of Sankar P et al. [22]. Most of them continued with their treatment protocols during the lockdown in contrast to the findings of Gautam V et al. [24] in which one-third of their study participants stopped taking the medicine. The overall compliance to medications was good with the majority not missing their medications during the lockdown, similar to the findings of Tao J et al. in which 78% of patients continued taking their regimen as prescribed and Alshareef R et al. [18] in which 88% patients took their medicines regularly during the lockdown. This compliance may be attributed to adequate counseling by the treating doctor and confidence in his prescription, which motivates the patients to buy medicines for a long time. This could partly be explained by the finding that the majority of patients in the present study didn’t run out of medicine ever during the lockdown. If the physician is having treatment inertia, they tend to call patients for frequent follow-ups, which has a negative effect on drug adherence [28].

Nearly 57% of our respondents who were on insulin were using the insulin syringes more than five times, which is in coherence with a previous study from India. Overall, needle reuse in India is much higher than in the rest of the world [29]. The principal reasons patients reuse needles are convenience and cost [18]. However, very few are aware of the fact that needle reuse is an established cause of increased pain during injection and lipohypertrophy [30]. Inaccessibility of needles and pens during the lockdown may be attributed as one plausible explanation for the increased repeated use. However, patients on insulin must be taught about the same during their clinical consultations.

Our study has certain strengths and limitations. The study was conducted in a super-specialty clinic, which ensures adequate treatment prescription, minimal treatment inertia, and good clinical practices while offering a clinical prescription. Data were collected using pre-validated tools, which make the results reliable. However, this is a single-center clinic-based study, which limits its generalizability. The picture can vary at primary or secondary health care centers and government health facilities where many medications are available free of cost under the National Programme for Prevention and Control of Cancer, Diabetes, Cardiovascular Diseases and Stroke (NPCDCS). Future studies should entail the assessment of people with diabetes taking treatment from different places for disease management through a community-based study to give a true picture. The responses related to changes in diet, physical activity, and stress are deemed to be affected by recall bias, which may not reflect a true picture. Telephonic interviews also leave scope for self-reporting bias, however, it was the best way to reach out to the patients during the pandemic. Lastly, the results may not be the same for people with a shorter duration of diabetes, as they may not have been adequately sensitized regarding diabetes self-care practices.

## Conclusions

In conclusion, our study disapproved our initial hypothesis that lockdown could have jeopardized the management of chronic diseases like diabetes. More time for self-care, adequate counseling about glycemic goals, and knowledge of self-monitoring of blood glucose levels have helped the majority of our patients in adopting a healthy lifestyle and achieve better glycemic control during the COVID-19 lockdown, which is expected to continue in the near future as well. The present study offers insight regarding the management of chronic diseases with minimal contact. We recommend the use of standard treatment guidelines for prescriptions, which should be followed by adequate counseling and regular monitoring. This can then lead to improved treatment compliance and hence better clinical outcomes to minimize the morbidity and mortality caused by the COVID-19 pandemic. Reinforced and frequent counseling will also help in improving diabetes self-management in case of similar pandemics and resulting restrictions in the future.
